# Characterizing dynamic functional connectivity subnetwork contributions in narrative classification with Shapley values

**DOI:** 10.1162/NETN.a.25

**Published:** 2025-09-19

**Authors:** Aurora Rossi, Yanis Aeschlimann, Emanuele Natale, Samuel Deslauriers-Gauthier, Peter Ford Dominey

**Affiliations:** COATI, Université Côte d’Azur, INRIA, CNRS, I3S, Sophia Antipolis, France; CRONOS, Inria Centre at Université Côte d’Azur, Sophia Antipolis, France; Université Bourgogne Europe, INSERM, CAPS UMR 1093, Dijon, France; Robot Cognition Laboratory, Marey Institute, Dijon, France

**Keywords:** fMRI, Dynamic functional connectivity, Narratives, Shapley values, Machine learning, Convolutional neural networks

## Abstract

Functional connectivity derived from functional magnetic resonance imaging (fMRI) data has been increasingly used to study brain activity. In this study, we model brain dynamic functional connectivity during narrative tasks as a temporal brain network and employ a machine learning model to classify in a supervised setting the modality (audio, movie), the content (airport, restaurant situations) of narratives, and both combined. Leveraging Shapley values, we analyze subnetwork contributions within Yeo parcellations (7- and 17-subnetworks) to explore their involvement in narrative modality and comprehension. This work represents the first application of this approach to functional aspects of the brain, validated by existing literature, and provides novel insights at the whole-brain level. Our findings suggest that schematic representations in narratives may not depend solely on preexisting knowledge of the top-down process to guide perception and understanding, but may also emerge from a bottom-up process driven by the temporal parietal subnetwork.

## Author Summary

This study investigates how different brain subnetworks contribute to processing narratives. We used a machine learning model to analyze fMRI data from participants listening to or watching narratives that varied in modality (audio or movie) and thematic content (airport or restaurant). Our model accurately classified these different narrative aspects, and by using Shapley values, we identified the subnetworks most crucial for each classification. Consistent with existing neuroscience knowledge, our findings highlight the distinct roles of these subnetworks in narrative comprehension. This study provides a powerful approach for investigating brain function across various domains.

## INTRODUCTION

Understanding the principles of representation and computation in the human brain, and developing corresponding predictive models, remains one of the great open challenges in neuroscience. fMRI provides a rich window into the dynamics of the whole human brain with a certain level of spatial and temporal resolution. From the beginning, human language processing has been a target of investigation with fMRI (Price, 2012). Experiments with words and sentences allowed the identification of language processing areas and networks at different levels of structure (Keller, Carpenter, & Just, 2001). More recently, evidence has emerged that language processing involves even broader recruitment across the brain, which might be obscured by time averaging and thresholding (Aliko, Wang, Small, & Skipper, 2023). This is consistent with studies that revealed how language recruits an extended frontotemporoparietal semantic system beyond the classic perisylvian language network (Binder & Desai, 2011; Jouen et al., 2015; Xu, Kemeny, Park, Frattali, & Braun, 2005). This has been demonstrated in the processing of narrative, full stories, which produce wide recruitment of whole brain networks for memory, visuospatial representation, and emotion (Jääskeläinen, Sams, Glerean, & Ahveninen, 2021; Silbert, Honey, Simony, Poeppel, & Hasson, 2014; Xu et al., 2005). Thus, narrative processing is a privileged context for the investigation of brain functional dynamics (Willems, Nastase, & Milivojevic, 2020). How can these functional dynamics be characterized? Analysis methods based on time averaging and subtraction tend to ignore the contribution of brain systems whose activity is variable and averaged out during thresholding. Functional connectivity analysis can be used to capture and characterize these dynamic interactions of brain regions over time (Preti, Bolton, & Van De Ville, 2017; Sizemore & Bassett, 2018). Temporal brain networks model the evolution of functional connectivity over time and thus have the desired properties of capturing the full brain dynamics that may be lost in time averaging and thresholding. Here, we exploit the representational richness of dynamic functional connectivity in temporal brain networks to characterize brain dynamics during narrative processing using machine learning.

In particular, we propose a simple machine learning model to classify in a supervised setting fMRI data collected during a narrative comprehension task. The model is mainly composed of a convolutional layer and a multilayer perceptron (MLP). It is trained to classify the modality of the narrative (audio or video), the content of the narrative (airport or restaurant situations), and these two together in a four-class classification. We use the model to investigate the importance of temporal dynamics in narrative processing and combined with the powerful explainability technique of Shapley values, we delve deeper into the model’s decision-making process. Specifically, we quantify the subnetwork contributions in the classification of two different parcellation methods (Yeo 7-subnetwork and 17-subnetwork) and this allows us to identify the most involved subnetworks in the narrative processing task. Our work is the first to apply this approach to functional aspects of the brain, validated by existing literature, and provides novel insights at the whole-brain level.

The results provide valuable insights, validated by existing research on narrative comprehension (Baldassano, Hasson, & Norman, 2018; Simony et al., 2016) and contribute to a broader understanding of how we process narratives. Our findings challenge the initial assumption that narrative comprehension relies solely on top-down activation of scripts, where prior knowledge, experiences, and expectations solely guide interpretation (Dubin & Bycina, 1991). The notable role of the temporal parietal subnetwork in content classification suggests a more nuanced model. This network is associated with bottom-up attentional control, implying that narrative processing might involve the assembly and integration of sensory information from the environment alongside top-down influences. This possibility aligns with the notion that schematic representations may not solely be driven by top-down activation but could be built upon bottom-up processing mediated by the temporal parietal subnetwork (Vossel, Geng, & Fink, 2014).

### Related Works

#### Classification of tasks from fMRI data.

Numerous studies have explored classifying tasks and subject characteristics (such as age and sex) from functional brain connectivity data using fMRI, primarily aiming to develop powerful architectures. Examples include the work by B.-H. Kim, Ye, and Kim (2021), where they propose a Spatio-Temporal Attention Graph Isomorphism Network model for high-accuracy prediction of seven tasks (memory, social, relational, motor, language, gambling, and emotion) alongside sex. Another approach by P. Kim et al. (2023) utilizes a transformer to classify age, sex, and cognitive intelligence, with an integrated gradient technique for interpreting sex classification results. The latter explainability technique is also employed in a parallel similar work by Ryali, Zhang, de Los Angeles, Supekar, and Menon (2024), where they classify sex using a simpler spatiotemporal deep neural network. Other papers by Huang, Xiao, and Wu (2021) and Saeidi et al. (2022) use a deep learning model, mainly composed of a convolutional neural network and a recurrent neural network, and a graph neural network, respectively, to classify the 7 tasks.

#### Narratives classification.

In contrast to the aforementioned papers, our work focuses on a more detailed classification domain, specifically the classification of modalities (movie, story) and the thematic content of the script (airport, restaurant). Baldassano et al. (2018) exemplify this approach, using a stochastic hidden Markov model to classify, based on the activation of a selection of regions of interest (ROIs) in the default attention networks, thematic content while also incorporating event alignment.

#### Shapley values in brain networks.

The use of Shapley values has become a popular approach to explain the predictions of machine learning models. In neuroscience, for instance, Amoroso et al. (2023) classify three conditions (Alzheimer’s disease, mild cognitive impairment, and healthy controls) based on brain structural connectivity data from MRI scans. They then leverage Shapley values to identify the most influential “patch” for classification. Another study by Kötter et al. utilizes Shapley ratings in macaque brain networks, employing a graph theory approach to analyze these networks. Here, the number of strongly connected components within a subgraph serves as the Shapley value function (Kötter et al., 2007). The most similar work to ours is by Li et al. (2020). They propose a new estimation method for Shapley values and apply it when classifying functional connectivity data from fMRI. In their example, they classify patient conditions (autism spectrum disorder or healthy) and compute the importance of different ROIs in classification, though they don’t delve into the neuroscientific interpretation of the results.

### Our Contribution

This study combines machine learning with explainable artificial intelligence (AI) to investigate the specific roles of brain subnetworks during tasks involving narratives. We leverage functional connectivity, extracted from fMRI data, and Shapley values to identify which brain subnetworks are most influential in classifying narrative modality (audio and movie), thematic content (airport and restaurant situation), and their combination. The fMRI data are segmented into 7 or 17 Yeo subnetworks using the Schaefer 100 element parcellation (Schaefer et al., 2018). Our machine learning model, composed of a convolutional neural network and MLP, achieves high accuracy and reveals the specific contributions of Yeo subnetworks in narrative processing. Importantly, the focus of our analysis is functional connectivity, rather than activation. This analysis, validated by neuroscientific interpretation aligned with existing literature, offers new insights into the functional roles of these subnetworks and the factor of time during narrative classification. Our work demonstrates the power of explainable AI in unveiling the complex interplay between brain activity and narrative comprehension. It not only helps to understand narrative processing but also paves the way for applying this approach to other areas of brain research.

## METHODS

### Model

Our model takes as input a temporal brain network. This network is a sequence of brain networks, each reflecting the brain’s functional connectivity at a specific time step (further details regarding the data processing are provided in the Experiments section). Mathematically, the temporal brain network can be represented as a three-dimensional tensor, denoted by *X* ∈ [−1, 1]^*R*×*R*×*T*^, where *R* represents the number of brain regions and *T* represents the number of time steps. In our case, *R* is 100 and *T* is 8.

**Figure 1.**
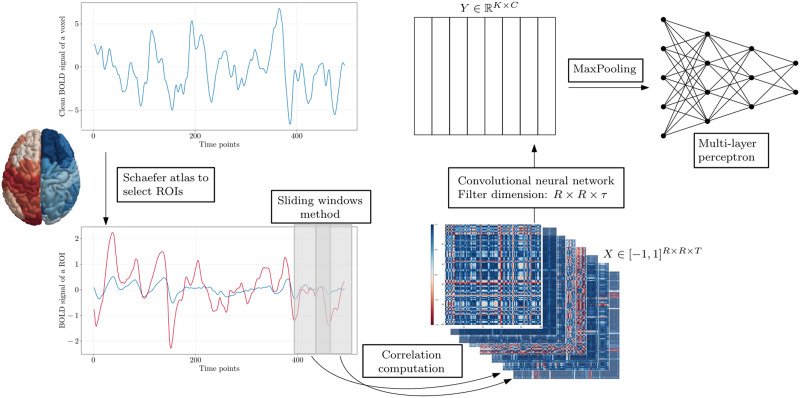
Pipeline from the extraction of temporal brain networks to the classification of the narrative aspects. The first step is the division of the brain into regions according to an atlas. The second step is the sliding window method, which individuates rectangular windows within which the Pearson correlation coefficient is computed between each pair of brain region time series. The output is then fed into the model, which consists of a convolutional layer, a max-pooling layer, and an MLP.

The model architecture consists of a single-layer three-dimensional convolutional neural network, followed by a max pooling layer and an MLP for classification. The convolution filter has size (*R*, *R*, *τ*) with no padding, where the two first dimensions match with those of the input. This design focuses on capturing temporal features within the brain network by restricting filter movement to the temporal axis. Max pooling is then applied to reduce the dimensionality of the extracted features. Finally, an MLP performs the classification task. A visual representation of the model architecture is provided in Figure 1.

Notably, when the filter size in the temporal dimension is set to 1 (*τ* = 1), the model becomes invariant to the specific order of time steps in the input data. An analysis of the model’s performance with different filter sizes is provided in the Appendix section.

Formally, given an input tensor *X* ∈ [−1, 1]^*R*×*R*×*T*^, the output of the convolutional layer is defined as

$$Y_{k,c}=\sigma {\left(X*W+b\right)}_{k,c}=\sigma \left(\sum_{i=1}^{R}\sum_{j=1}^{R}\sum_{p=1}^{\tau }X_{i,j,k+p-1}\cdot W_{i,j,p,c}+b_{k,c}\right)$$

where *Y* ∈ ℝ^*K*×*C*^ is the output tensor, *W* ∈ ℝ^*R*×*R*×*τ*×*C*^ is the learnable filter tensor, *b* ∈ ℝ^*K*×*C*^ is the bias matrix, and *C* is the number of output channels. The operations, + and *σ*, which represent the ReLU(*x*) = max ({0, *x*}) activation function, are applied component-wise. The output tensor is then passed through a max-pooling layer so that the output vector *Z* ∈ ℝ*^C^* is defined as

$$Z=\max_{k}Y\left[k,c\right].$$

Finally, the output passed through an MLP of three fully connected layers with ReLU activation functions. A fully connected layer can be defined as *V* =*σ*(*W* ⋅ *Z* +*b*) where *V* is the output of the fully connected layer, *W* is the weight matrix, and *b* is the bias vector.

### Shapley Values

Shapley values were introduced by Lloyd Shapley in 1951 in the context of cooperative game theory (Shapley, 1951). They quantify the contribution of each player in a coalition game. Recently, they have been adopted in machine learning to explain the predictions of models. Shapley values can be calculated using different methods including sampling or exact computation for smaller player sets (Lundberg & Lee, 2017). In our case, we leverage Shapley values to understand the influence of specific brain subnetworks on the prediction of our model. Because of the limited number of brain subnetworks defined by the 7 Yeo parcellation method (Thomas Yeo et al., 2011), we can compute the exact Shapley values. The exact Shapley value of a brain subnetwork *i* is defined as

$$\phi_{i}\left(v\right)=\sum_{S\subseteq N\backslash \left\{i\right\}}\frac{|S|!\left(|N|-|S|-1\right)!}{|N|!}\left(v\left(S\cup \left\{i\right\}\right)-v\left(S\right)\right)$$ (1)

where *N* is the set of brain subnetworks and *v* is the accuracy of our model when considering the set *S* of brain subnetworks. To isolate the influence of specific subnetworks within the temporal brain network *X*, we mask other subnetworks by setting to zero the rows and columns of the adjacency matrix corresponding to regions within the subnetwork. Zero was chosen as masking value because it represents the mean connectivity value following Z-normalization of the data. The Shapley value *ϕ_i_*(*v*) is the average marginal contribution of the brain subnetwork *i* over all possible combinations of brain subnetworks, the higher the Shapley value, the more important the brain subnetwork is for the prediction of the model. For the 17 Yeo subnetwork parcellation, the exact computation of Shapley values becomes computationally expensive. Therefore, we employ a sampling method that approximates the Shapley values using the same formula but instead of summing over all possible subnetwork combinations, we sample a large number of combinations (100 samples in our case) to approximate the average marginal contribution.

### Experiments

Experiments were performed to determine if the temporal brain networks can be used to discriminate brain functional connectivity patterns in response to audio versus movie narratives, airport versus restaurant situations, and the combination of these two dimensions. We trained a machine learning model in a supervised setting to classify these aspects and used Shapley values to interpret the model’s decisions.

#### Data.

##### Dataset.

Our analysis used fMRI data from the study of Baldassano et al. (2018) archived as part of the Narratives dataset created by Nastase et al. (https://openneuro.org/datasets/ds002345/versions/1.1.4; Nastase et al., 2020). The Baldassano dataset (https://openneuro.org/datasets/ds001510/versions/1.0.1) includes brain activity recordings from 31 participants engaged in a narrative task. In this task, each subject is exposed to sixteen 3-min stories (four per run over four runs) from two different scripts (eating at a restaurant or going through the airport). While the stories within each category share a similar high-level sequence of events, there are variations in the specific details of these events. Each run presents two movies and two audio stories, for a total of eight movies and eight audio segments over the course of the experiment. The dataset is balanced in terms of the number of samples per modality and content.

##### Preprocessing.

The fMRI data has a spatial resolution of 91 × 91 × 109 voxels in the *x, y*, and *z* axes, respectively, for a total of 902,629 voxels. Each voxel measures 2 × 2 × 2 mm. The repetition time is 1.5 s, for a total of 490 time points and a total duration of 12 min per run approximatively.

Preprocessing involved transforming the blood-oxygen-level-dependent (BOLD) signals from each voxel into temporal graphs. We implemented a pipeline to reduce motion artifacts by performing linear regression on the movement parameters. Additionally, a band-pass filter (0.01−0.08 Hz) was applied to remove noise arising from respiration and cardiac pulsations (Van Dijk et al., 2010).

To define the network nodes, we employed the Schaefer et al. (2018) brain atlas (after having put the data in the MNI152 space), parcellating the brain into 100 ROIs based on anatomical and functional criteria. ROIs were created by averaging the BOLD time series of voxels within gray matter regions. We then utilized a sliding window approach with 30-s windows and 7.5 s overlap to divide the data into time steps. The Pearson correlation coefficient was computed between each pair of ROI time series within each window, with the resulting correlation value assigned as the weight of the edge connecting the corresponding ROI nodes. This process yielded an adjacency matrix for each time window, and the sequence of these matrices formed the temporal brain networks (see Figure 1). The resulting adjacency matrices were Z-normalized, centering the data around zero with a standard deviation of one. This normalization step reduces the influence of outliers and helps ensure that the selection of masking values does not introduce bias. For example, without normalization, if the average correlation between two regions is approximately 0.40 for class one and 0.45 for class two, inputting a correlation value of 0.00, significantly lower than both averages, could bias the model toward predicting class one, as lower correlations are more commonly associated with that class.

#### Experimental setting.

The experiments were conducted on a workstation equipped with a single NVIDIA Quadro RTX 8000 graphics card. We utilized the Julia programming language for the workflow, from network creation starting from the clean signal to the model development (Bezanson, Edelman, Karpinski, & Shah, 2017). The Flux.jl library was used for neural network implementation and the Makie.jl library was used for visualization (Danisch & Krumbiegel, 2021; Innes et al., 2018). The source code is available at the following GitHub repository: https://github.com/aurorarossi/fMRINarrativeClassification.

##### Hyperparameters.

The hyperparameters were chosen based on empirical observations. The convolutional filter *τ* parameter was set to 4 for the modality classification task and 8 for the content and the combined classification task (see the Appendix for more details). The number of output channels was set to 128 for all the tasks. The MLP had two hidden layers with 64 and 32 units each with a ReLU activation function. The output dimension of the MLP was set to 2 for the modality classification task, 2 for the content classification task, and 4 for the combined classification task. The data comprises 16 narratives, with connectivity analyzed within eight temporal windows per story, yielding a dataset of dimensions 100 × 100 × 8 for each participant (representing a temporal brain). With 31 participants, each exposed to 16 narratives, the overall dataset dimensions are 100 × 100 × 8 × 31 × 16. To contextualize model complexity:

▪  For *τ* = 4: Convolution: 100 × 100 × 128 × 4; MLP layers: 128 × 64 + 64 × 32 + 32 × 2,
▪  For *τ* = 8: Convolution: 100 × 100 × 128 × 8; MLP layers: 128 × 64 + 64 × 32 + 32 × 4.

This analysis reveals that the model complexity remains within an order of magnitude of the dataset size, ensuring a reasonable balance between model capacity and data availability.

##### Training.

Given the limited size of the dataset, we employed a batch size of 1 during training. We used the Adam optimizer with a learning rate of 0.0001. The training process lasted for 20 epochs. The choice of 20 epochs was determined through experiments to achieve a good balance between training time and model performance. For the loss function, we used either logit binary cross-entropy or logit cross-entropy depending on the number of classes in the task. To ensure robustness against potential variations due to model initialization, we retrain the model 15 times with different random splits of the data (80% training, 20% testing). During each iteration, we compute both the Shapley values and the model’s accuracy. Finally, we report the mean and standard deviation to account for variability for the accuracy, and for Shapley values of each subnetwork, we present the mean values along with error bars representing the standard deviation. This approach ensures a comprehensive understanding of the model’s performance, the contribution of individual brain subnetworks to its classifications, and the robustness of these findings across model initializations.

## RESULTS

In this section, we describe the results of our experiments. We present the performance of the model on three classification tasks:

▪  **Modality classification:** This task focuses on classifying the brain network based on the modality of the stimuli, audio or movie.
▪  **Content classification:** The model classifies the brain network based on the content of the stimuli, airport, or restaurant situations.
▪  **Combined modality and content classification:** This task evaluates the model’s ability to jointly classify both the modality and the content of the stimuli.

The results in Table 1 show that the model performs well on the modality classification task, achieving an accuracy of 95.69% ± 1.56%. While still a good performance considering the complexity, the model’s accuracy on the content classification task was at 79.37% ± 2.59%. This difference might be attributed to the inherent difficulty of content classification compared with modality identification. Furthermore, the combined modality and content classification task resulted in an accuracy of 70.34% ± 4.34%. Notably, the model displayed consistent performance across all metrics.

**Table 1.** Performance metrics of the model across modality, content, and combined classification tasks

	Modality	Content	Both modality and content
Accuracy	95.69% ± 1.56%	79.37% ± 2.59%	70.34% ± 4.34%
Precision	96.24% ± 1.75%	79.79% ± 3.86%	71.77% ± 8.44%
Recall	95.14% ± 2.57%	79.09% ± 4.12%	70.35% ± 7.80%
F1 score	95.66% ± 2.08%	79.30% ± 3.99%	70.92% ± 8.96%
Accuracy permuting network windows	86.46% ± 2.67%	57.71% ± 5.98%	46.25% ± 6.11%
Accuracy permuting time series	49.44% ± 3.95%	48.61% ± 3.44%	23.47% ± 3.29%
Static functional connectivity accuracy	87.57% ± 2.31%	75.20% ± 2.95%	64.03% ± 3.31%

The row “Accuracy permuting network windows” reflects the model’s performance when brain network time steps are permuted, while “Accuracy permuting time series” shows performance when the time series are permuted prior to constructing the network. The last row reports the model’s performance using static functional connectivity matrices.

To assess the importance of the time dimension in classification tasks, we performed two types of permutation: First, we shuffled the time series before constructing the network; second, we shuffled the network windows while keeping the time steps within each window intact, followed by retraining the model. As expected, shuffling the entire time series led to significantly lower accuracy compared with shuffling the network windows.

When shuffling the network windows, the time evolution within each window remains consistent with the original data, essentially creating a block permutation. This means that while the order of windows is altered, the temporal relationships within each window are preserved. In contrast, shuffling the entire time series disrupts the sequential flow, completely dismantling its temporal structure. This disruption impacts both content and modality classification, as both rely heavily on the temporal context of the brain activity being analyzed.

In the case of shuffling network windows, the results show a notable drop in accuracy compared with the unshuffled data: 9% for modality classification, a substantial 22% for content classification, and 24% for the combined task. The performance decrease is more pronounced in content and combined classification tasks compared with modality classification. In the Discussion section, we will explore how to interpret the results of this permutation tests with respect to the model parameters.

For static functional connectivity matrices, the results remain relatively high: 87.57% ± 2.31% for modality classification, 75.20% ± 2.95% for content classification, and 64.03% ± 3.31% for the combined task. While these results indicate that static features provide valuable information, the performance is notably lower compared with when the model incorporates dynamic time series data. This suggests that while static functional connectivity offers useful insights, integrating temporal information significantly improves the model’s ability to accurately classify brain activity.

**Figure 2.**
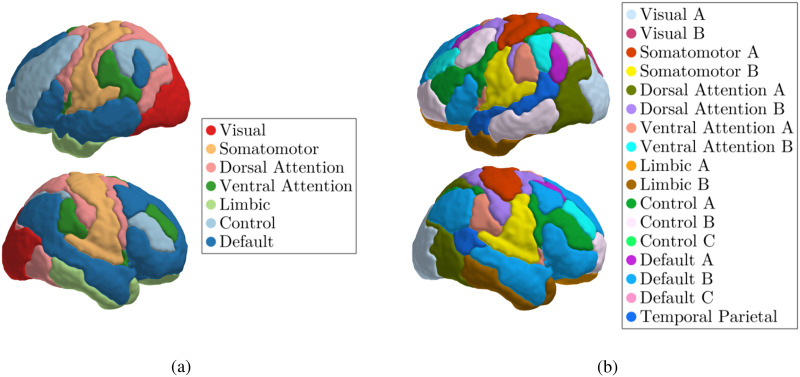
Yeo parcellations used in the Shapley value analysis. The 7-subnetwork parcellation is shown on the left (A), while the 17-subnetwork parcellation is shown on the right (B).

To gain deeper insights into how the model leverages brain activity for classification, we employed Shapley values. Here, we focus on subnetworks defined by the Yeo parcellation method (Thomas Yeo et al., 2011), specifically the 7-subnetwork and 17-subnetwork parcellations. Visualizations of these parcellations are provided in Figure 2. Black and white compatible versions of these figures can be found in the Appendix.

**Figure 3.**
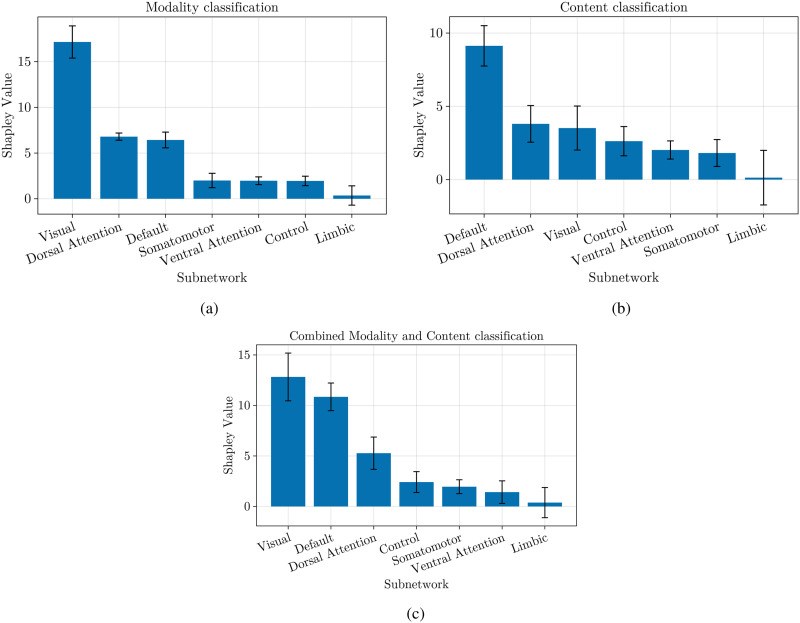
This figure shows the contribution of Yeo 7 subnetworks computed with Shapley values for classifying narrative using a machine learning model. The bars represent the average contribution of each subnetwork to the model’s predictions, with higher values indicating greater influence. The error bars represent the standard deviation of the Shapley values.

Figure 3 presents the Shapley values for the 7-subnetwork parcellation. In the modality classification task, the visual subnetwork emerges as the most influential (Figure 3A). This aligns with the intuitive notion that processing visual information plays a key role in distinguishing modalities. For the content classification task, the high value of the default mode subnetwork suggests its influence in understanding the meaning and content of the stimuli as suggested by previous studies (Baldassano et al., 2018; Simony et al., 2016; Figure 3B). Finally, the combined classification task reveals the importance of both the visual and default mode networks (Figure 3C), suggesting that the model utilizes a combination of visual features and higher order processing for accurate content and modality classification.

**Figure 4.**
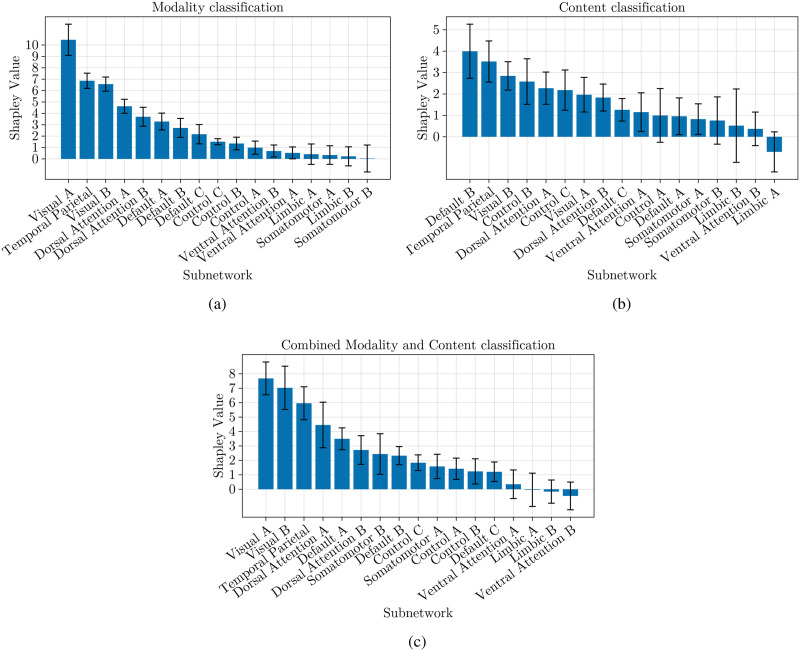
This figure shows the contribution of Yeo 7 subnetworks computed with Shapley values for classifying narrative using a machine learning model. The bars represent the average contribution of each subnetwork to the model’s predictions, with higher values indicating greater influence. The error bars represent the standard deviation of the Shapley values.

Figure 4 presents the Shapley values for the 17-subnetwork parcellation. In the modality classification task, the visual A and B, and temporal parietal subnetworks emerge as the most influential (Figure 4A). For the content classification task, the default B subnetwork and the temporal parietal also play crucial roles (Figure 4B). Finally, the combined classification task reveals the importance of the visual A and B and temporal parietal subnetworks (Figure 4C).

**Figure 5.**
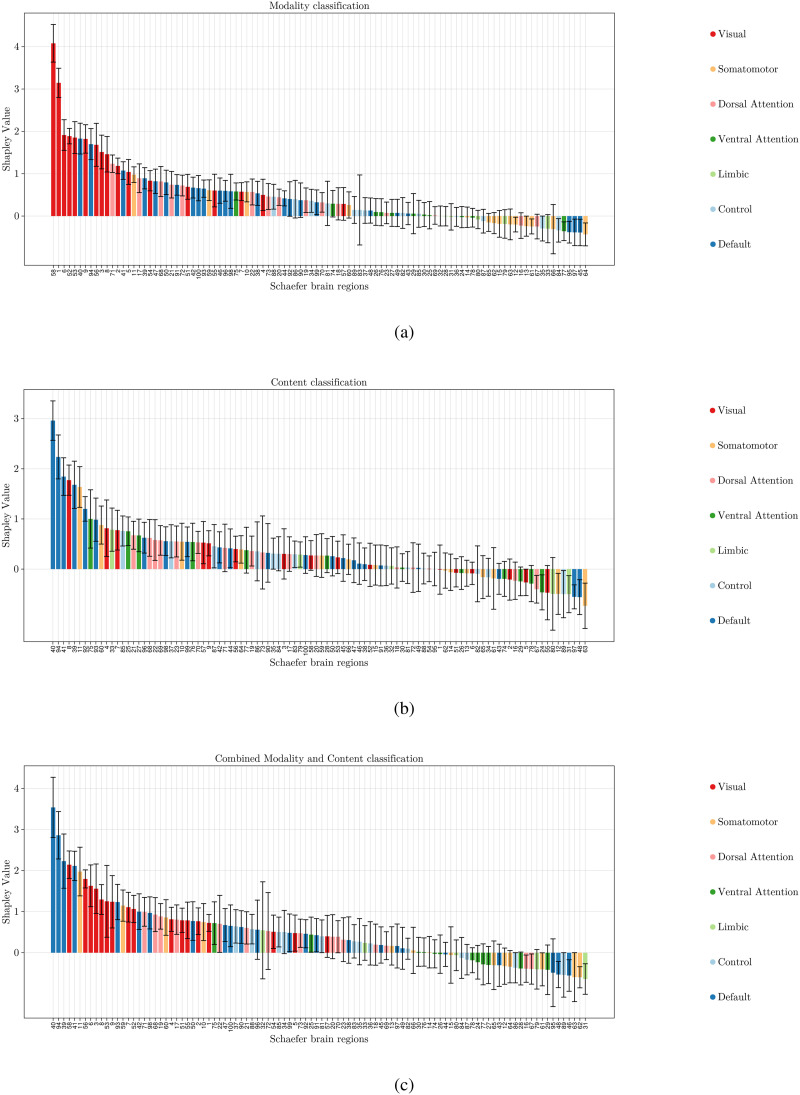
This figure shows the contribution of 100 Schaefer subnetworks, computed using Shapley values, for classifying narratives with our machine learning model. The bars represent the average contribution of each subnetwork to the model’s predictions, with higher values indicating greater influence. The error bars denote the standard deviation of the Shapley values. Additionally, the color of each bar corresponds to one of the seven subnetworks in the Yeo 7 parcellation.

**Figure 6.**
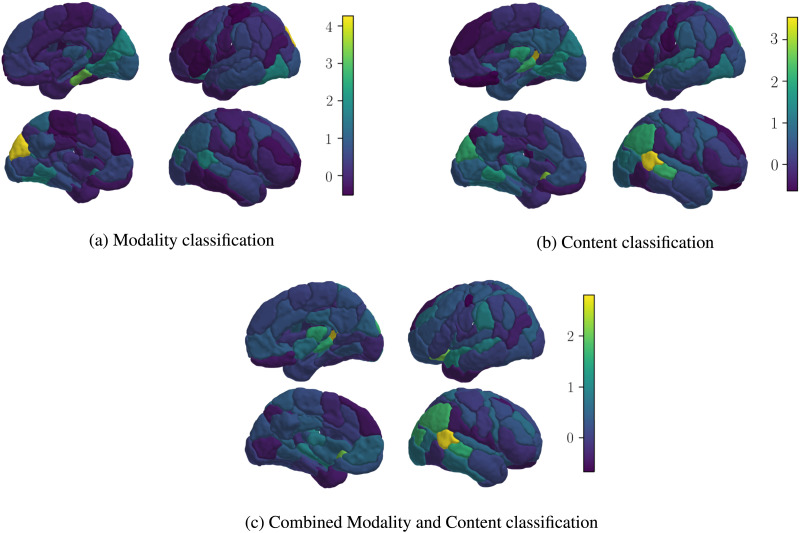
This figure presents the results of Figure 5 visualized on the surface of the brain. The contribution of each of the 100 Schaefer subnetworks, calculated using Shapley values, is mapped onto the corresponding brain regions. The color of each brain region reflects its average contribution to the model’s predictions, with lighter colors indicating greater influence.

Figure 5 and Figure 6 show the Shapley scores for the 100 parcellations of the Schaefer subnetworks, which are consistent with the results of the Yeo parcellations. The visual network emerges as the most significant for modality classification. For content classification, the default mode network is dominant, while for combined classification, both networks are important.

## DISCUSSION

This work investigated the neural basis of narrative processing using a machine learning model that classifies narrative aspects (modality, content, combined) based on functional connectivity networks derived from fMRI data. The model’s performance aligned with expectations: higher accuracy for modality classification, which is a simpler task because it relies on sensory information, compared with content classification which requires a deeper understanding of the narrative.

Permuting time steps in the temporal brain network significantly reduced classification accuracy, particularly in content and combined tasks, with a substantial drop of 20% as shown in Table 1. This occurred when the model employed a convolutional filter with a temporal dimension *τ* = 8, indicating that the model utilized temporal relationships between adjacent time windows for optimal classification. Conversely, with *τ* = 1, the model became invariant to the specific order of time steps, processing each temporal window independently. This resulted in a more modest accuracy decrease (see Figure 1 in the Appendix), suggesting that content-sensitive features are already captured within individual windows. This is expected given the windowed construction of features, where Pearson correlations are computed within each window, inherently encoding some temporal information within that window. Therefore, the model with *τ* = 1 emphasizes intrawindow feature extraction, while the model with *τ* = 8 relies on the importance of interwindow relationships. These findings highlight how model design and training influence classification accuracy, while also suggesting that a substantial amount of temporal information is inherently captured within each window.

To delve deeper into the model’s decision-making process, we employed Shapley values, a powerful explainable AI technique that quantifies subnetwork contributions. While techniques like Grad-CAM and Eigen-CAM provide valuable insights in image data, where spatial localization is crucial, they are less applicable in our context (Muhammad & Yeasin, 2020; Selvaraju et al., 2020). The rows and columns of the functional connectivity matrix capture the correlations among respective brain regions, but without a clear invariance relationship motivating the use of convolutional kernels across regions. Conversely, it is natural to assume the existence of time-invariant features for our task. This assumption is validated by our results, which demonstrate performance degradation when the sequence of connectivity graphs is randomly shuffled across time. This motivation supports the use of our convolutional neural network operating on the temporal dimension of the data. Class activation map techniques would highlight salient time steps determining the output but would not provide insights into the relevant brain regions. In contrast, our use of Shapley coefficients allows us to control the masking of specific subnetworks, enabling an analysis of individual brain regions’ contributions to the model’s predictions while accommodating our temporal data setup.

Our findings revealed that in the 7-subnetwork analysis, the visual subnetwork is the key for modality classification, reflecting the intuitive notion that visual processing is essential for distinguishing between movies and audio stories. In content classification, the default mode subnetwork emerged as the most influential, suggesting its essential function in understanding the meaning and content of the stimuli. This aligns with existing research that has highlighted the default mode subnetwork’s involvement in higher order cognitive functions, such as narrative comprehension (Baldassano et al., 2018; Simony et al., 2016). The combined classification task emphasized the importance of both visual and default mode networks, as expected.

Similar to the 7-subnetwork analysis, the default mode B subnetwork was most influential for content classification. Interestingly, the temporal parietal subnetwork also played a notable role. This finding is a step further to answer the open question raised by the Baldassano et al. (2018) study. They proposed that schematic representations in the brain might not solely rely on top-down activation of scripts in the medial prefrontal cortex. They suggested these representations could serve as building blocks for a complete narrative script formed through a bottom-up process. Our observation of a considerable Shapley value for the temporal parietal subnetwork, which is known to be also associated with bottom-up attentional control, aligns with this possibility. Finally, the combined classification task again highlighted the importance of visual and temporal parietal networks.

### Limitations and Future Works

It is important to acknowledge that the primary limitation of this study is the size of the dataset used. This may limit the generalizability of our findings to other populations or narrative stimuli. Future research could address this by employing larger datasets, if available. Additionally, exploring the generalizability of these findings across diverse datasets would be valuable. Within the context of the current dataset size, future work could delve deeper into other aspects of narrative processing. One potential direction is to investigate the impact of individual differences in narrative comprehension. For instance, research could explore how factors such as age, reading experience, or cultural background might influence how individuals process narratives based on brain network activity. In addition, it would be beneficial to explore the model’s decision-making process in more detail. Analyzing the learned weights of our neural architectures could provide complementary insights to those obtained from Shapley scores, which focus on model predictions. This approach could provide a clearer understanding of how specific brain regions contribute to the classification task. Future work could explore the temporal dynamics of narrative processing by examining the role of specific time windows in the classification task. Masking entire time steps and assessing the effect of window size on classification performance may shed light on how temporal information is integrated to understand narratives. Finally, computing Shapley values for connections between regions (edges, entries of the three-dimensional tensor) rather than for regions themselves presents an interesting alternative. In this interpretation, the “players” shift from individual regions to the relationships between them, providing insight into the most important connections for classification performance.

### Conclusion

Overall, our work demonstrates the potential of combining machine learning models with explainable AI techniques like Shapley values to understand the role of brain subnetworks during narrative processing. Our findings not only contribute to a deeper understanding of how the brain processes narratives but also showcase the broader applicability of this approach. In tasks where the role of specific brain regions remains unclear, this methodology can provide valuable new insights. By highlighting subnetwork contributions through Shapley values, we can generate novel hypotheses about the functional roles of these regions. In our case, the model’s performance aligns with existing literature on narrative comprehension, validating the approach. Importantly, this research validates an alternative and complementary method for investigating brain function in human cognition, which involves functional connectivity. This successful validation paves the way for further exploration of brain networks not only in higher order cognition, motor tasks, and emotional processing but also in any domain where the neural basis remains partially understood.

## ACKNOWLEDGMENTS

A.R. and E.N. would like to thank Pierluigi Crescenzi for the discussion on the explainability technique used in the paper. This work has been supported by the French government, through the UCA DS4H Investments in the Future project managed by the National Research Agency (ANR) with the reference number ANR-17-EURE-0004 and the ANR France Relance project. It has also been supported by the French government, through the 3IA Côte d’Azur Investments in the project managed by the ANR with the reference number ANR-23-IACL-0001. The authors are grateful to the OPAL infrastructure from Université Côte d’Azur for providing resources and support.

## SUPPORTING INFORMATION

Supporting information for this article is available at https://doi.org/10.1162/NETN.a.25.

## AUTHOR CONTRIBUTIONS

Aurora Rossi: Conceptualization; Data curation; Formal analysis; Investigation; Methodology; Resources; Software; Validation; Visualization; Writing – original draft; Writing – review & editing. Yanis Aeschlimann: Data curation. Emanuele Natale: Conceptualization; Investigation; Methodology; Project administration; Supervision; Writing – review & editing. Samuel Deslauriers-Gauthier: Conceptualization; Project administration; Supervision; Writing – review & editing. Peter Ford Dominey: Conceptualization; Project administration; Supervision; Writing – review & editing.

## FUNDING INFORMATION

Aurora Rossi, Agence Nationale de la Recherche (https://dx.doi.org/10.13039/501100001665), Award ID: ANR-17-EURE-0004.

## Supplementary Material



## TECHNICAL TERMS

Temporal brain network: A sequence of functional connectivity graphs representing how brain region interactions evolve over time.
Multilayer perceptron: A feedforward neural network composed of multiple fully connected layers.
Shapley values: A method to quantify each player’s contribution to the total payoff in a coalition game.
Explainable AI: Techniques that make machine learning model decisions interpretable without compromising prediction performance.
Model accuracy: The proportion of correct predictions made by a model on a classification task.
Classification task: A supervised learning setup where the goal is to assign input to predefined categories or classes.
Coalition game: A game theory model where players cooperate in groups (coalitions) for mutual gain.
Z-normalization: Standardizing data so it has a mean of zero and standard deviation of one to ensure comparability.
Convolution: Operation using filters to extract local patterns in data, commonly used in deep learning for image processing.
Epoch: One full pass through the training dataset during model learning, used to update model’s weights.
