# Supplementary material for: Characterizing dynamic functional connectivity subnetwork contributions in narrative classification with Shapley values
Source: Netw Neurosci. 2025 Sep 19;9(3):1138–53. doi: 10.1162/NETN.a.25 (PMC12548664; doi:10.1162/NETN.a.25)
Supplement: Supplementary file 1 [file netn-9-3-1138-s001.pdf]

1 Appendix

1.1 Choice of parameter τ

The following figure shows the evolution of the model's accuracy as a function of the third dimension of the convolutional filter (i.e. τ). For the modality classification, we set $\tau = 4$, since model performance seems not to increase significantly beyond this value (Figure 1a). For the content and combined classification, we set $\tau = 8$ since the model performance seems the best for this value (Figure 1b and Figure 1c). It is important to highlight that when $\tau = 8$ the convolution behaviour is similar to the one of dense layer.

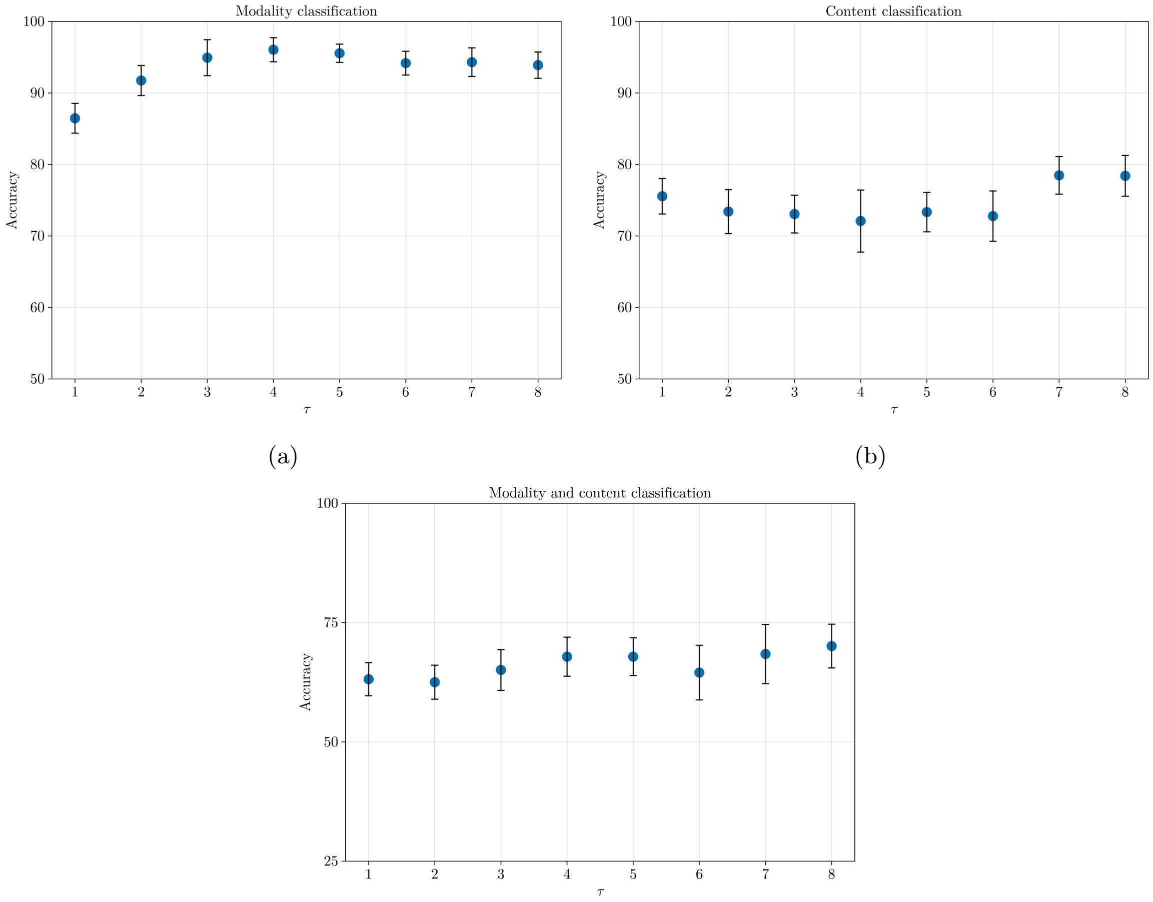

(a) (b) (c)

Figure 1: Model's accuracy as a function of the third dimension of the convolutional filter.

1.2 Desikan parcellation

The following figure shows the Shapley values for the Desikan parcellation in Figure 2. The Shapley values are calculated for the modality classification (Figure 2a), content classification (Figure 2b), and combined

classification (Figure 2c). Shapley values were calculated for 70 brain regions, showing that area 22 and 57, associated with left and right pericalcarine cortex or primary visual cortex, has the highest value in modality classification. This region is is primarily responsible for processing visual information. This is followed by regions 12 and 65, which are associated with the lateral occipital cortex and superior parietal cortex, respectively. These regions are involved in visual processing and spatial attention. In content classification, the right and left banks of the superior temporal sulcus stand out. These regions serve as hubs for social perception and cognition, including recognition of faces and human movement, as well as understanding actions, mental states and language. In addition, region 31, the superior temporal gyrus, remains important, consistent with previous findings. In the combined classification task, the middle temporal gyrus, the pericalcarine cortex and the superior temporal sulcus emerge as the most involved regions.

Table 1: Correspondence between Desikan labels and regions.

ID	Desikan Labels
1	L white matter
2	L Banks superior temporal sulcus
3	L caudal anterior cingulate cortex
4	L caudal middle frontal gyrus
5	L corpus calosum
6	L cuneus cortex
7	L entorhinal cortex
8	L fusiform gyrus
9	L inferior parietal cortex
10	L inferior temporal gyrus
11	L isthmus cingulate cortex
12	L lateral occipital cortex
13	L lateral orbitofrontal cortex
14	L lingual gyrus
15	L medial orbitofrontal cortex
16	L middle temporal gyrus
17	L parahippocampal gyrus
18	L paracentral lobule
19	L pars opercularis
20	L pars orbitalis
21	L pars triangularis
22	L pericalcarine cortex
23	L postcentral gyrus
24	L posterior cingulate cortex
25	L precentral gyrus
26	L precuneus cortex
27	L rostral anterior cingulate cortex
28	L rostral middle frontal gyrus
29	L superior frontal gyrus
30	L superior parietal cortex
31	L superior temporal gyrus
32	L supramarginal gyrus
33	L frontal pole
	Continued on next page

ID	Desikan Labels
34	L temporal pole
35	L transverse temporal cortex
36	R white matter
37	R Banks superior temporal sulcus
38	R caudal anterior cingulate cortex
39	R caudal middle frontal gyrus
40	R corpus calosum
41	R cuneus cortex
42	R entorhinal cortex
43	R fusiform gyrus
44	R inferior parietal cortex
45	R inferior temporal gyrus
46	R isthmus cingulate cortex
47	R lateral occipital cortex
48	R lateral orbitofrontal cortex
49	R lingual gyrus
50	R medial orbitofrontal cortex
51	R middle temporal gyrus
52	R parahippocampal gyrus
53	R paracentral lobule
54	R pars opercularis
55	R pars orbitalis
56	R pars triangularis
57	R pericalcarine cortex
58	R postcentral gyrus
59	R posterior cingulate cortex
60	R precentral gyrus
61	R precuneus cortex
62	R rostral anterior cingulate cortex
63	R rostral middle frontal gyrus
64	R superior frontal gyrus
65	R superior parietal cortex
66	R superior temporal gyrus
67	R supramarginal gyrus
68	R frontal pole
69	R temporal pole
70	R transverse temporal cortex

1.3 Schaefer parcellation

Table 2: Correspondence between Schaefer labels and regions.

ID	Schaefer Labels
1	LH Vis 1
2	LH Vis 2

Continued on next page

ID	**Schaefer Labels**
3	LH Vis 3
4	LH Vis 4
5	LH Vis 5
6	LH Vis 6
7	LH Vis 7
8	LH Vis 8
9	LH Vis 9
10	LH SomMot 1
11	LH SomMot 2
12	LH SomMot 3
13	LH SomMot 4
14	LH SomMot 5
15	LH SomMot 6
16	LH DorsAttn Post 1
17	LH DorsAttn Post 2
18	LH DorsAttn Post 3
19	LH DorsAttn Post 4
20	LH DorsAttn Post 5
21	LH DorsAttn Post 6
22	LH DorsAttn PrCv 1
23	LH DorsAttn FEF 1
24	LH SalVentAttn ParOper 1
25	LH SalVentAttn FrOperIns 1
26	LH SalVentAttn FrOperIns 2
27	LH SalVentAttn PFCl 1
28	LH SalVentAttn Med 1
29	LH SalVentAttn Med 2
30	LH SalVentAttn Med 3
31	LH Limbic OFC 1
32	LH Limbic TempPole 1
33	LH Limbic TempPole 2
34	LH Cont Par 1
35	LH Cont PFCl 1
36	LH Cont pCun 1
37	LH Cont Cing 1
38	LH Default Temp 1
39	LH Default Temp 2
40	LH Default Par 1
41	LH Default Par 2
42	LH Default PFC 1
43	LH Default PFC 2
44	LH Default PFC 3
45	LH Default PFC 4
46	LH Default PFC 5
47	LH Default PFC 6

Continued on next page

ID	Schaefer Labels
48	LH Default PFC 7
49	LH Default pCunPCC 1
50	LH Default pCunPCC 2
51	RH Vis 1
52	RH Vis 2
53	RH Vis 3
54	RH Vis 4
55	RH Vis 5
56	RH Vis 6
57	RH Vis 7
58	RH Vis 8
59	RH SomMot 1
60	RH SomMot 2
61	RH SomMot 3
62	RH SomMot 4
63	RH SomMot 5
64	RH SomMot 6
65	RH SomMot 7
66	RH SomMot 8
67	RH DorsAttn Post 1
68	RH DorsAttn Post 2
69	RH DorsAttn Post 3
70	RH DorsAttn Post 4
71	RH DorsAttn Post 5
72	RH DorsAttn PrCv 1
73	RH DorsAttn FEF 1
74	RH SalVentAttn TempOccPar 1
75	RH SalVentAttn TempOccPar 2
76	RH SalVentAttn FrOperIns 1
77	RH SalVentAttn Med 1
78	RH SalVentAttn Med 2
79	RH Limbic OFC 1
80	RH Limbic TempPole 1
81	RH Cont Par 1
82	RH Cont Par 2
83	RH Cont PFCl 1
84	RH Cont PFCl 2
85	RH Cont PFCl 3
86	RH Cont PFCl 4
87	RH Cont Cing 1
88	RH Cont PFCmp 1
89	RH Cont pCun 1
90	RH Default Par 1
91	RH Default Temp 1
92	RH Default Temp 2

Continued on next page

ID	Schaefer Labels
93	RH Default Temp 3
94	RH Default PFCv 1
95	RH Default PFCv 2
96	RH Default PFCdPFCm 1
97	RH Default PFCdPFCm 2
98	RH Default PFCdPFCm 3
99	RH Default pCunPCC 1
100	RH Default pCunPCC 2

1.4 Inter-intra subject variability

Intra-subject standard deviation (SD):We calculated the intra-subject standard deviation by first computing the standard deviation of accuracy for each individual subject. These individual standard deviations were then averaged across all subjects. The intra-subject standard deviation is given by:

$$SD = \frac{1}{N}\sum_{i=1}^{N} SD_i$$

where N is the total number of subjects and SD_i is computed as follows:

$$SD_i = \sqrt{\frac{1}{M-1}\sum_{k=1}^{M}(Accuracy_{i,k} - MeanAccuracy_i)^2}$$

Here $Accuracy_{i,k}$ is represents the accuracy for the k-th sample of subject i, $MeanAccuracy_i$ is the mean accuracy for subject i, and M is the number of samples for each subject (16).

Inter-subject standard deviation (SD):The inter-subject standard deviation was calculated by taking the standard deviation of the mean accuracy values across all subjects:

$$SD = SD(\frac{1}{N}\sum_{j=1}^{N} MeanAccuracy_j)$$

where $Accuracy_j$ is the mean accuracy for subject j and N is the total number of subjects.

Results:

For modality classification:

- Intra-subject standard deviation: 16.90%
- Inter-subject standard deviation: 4.91%
- Total variability: 21.68%

For content classification:

- Intra-subject standard deviation: 37.05%
- Inter-subject standard deviation: 8.04%
- Total variability: 38.02%

For combined classification:

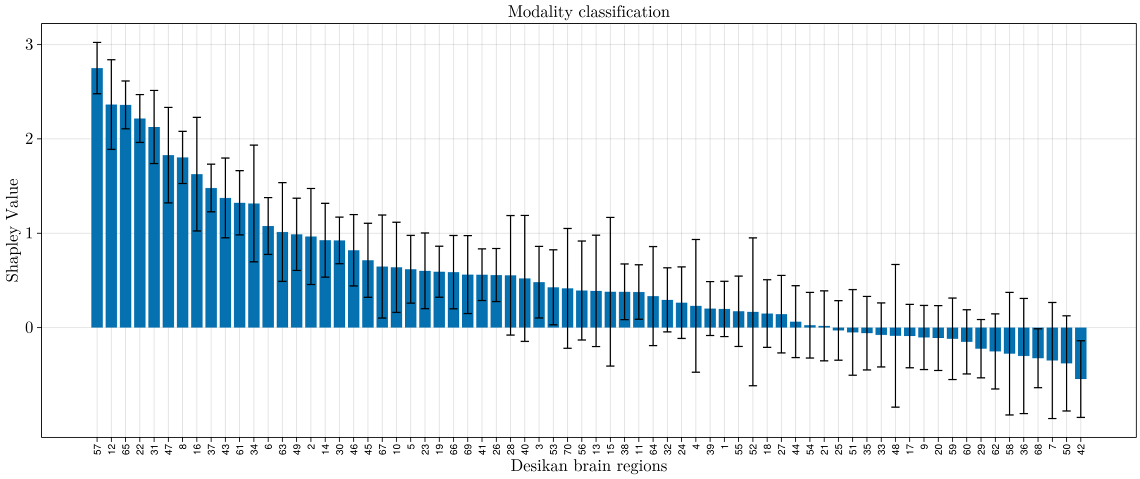

(a)

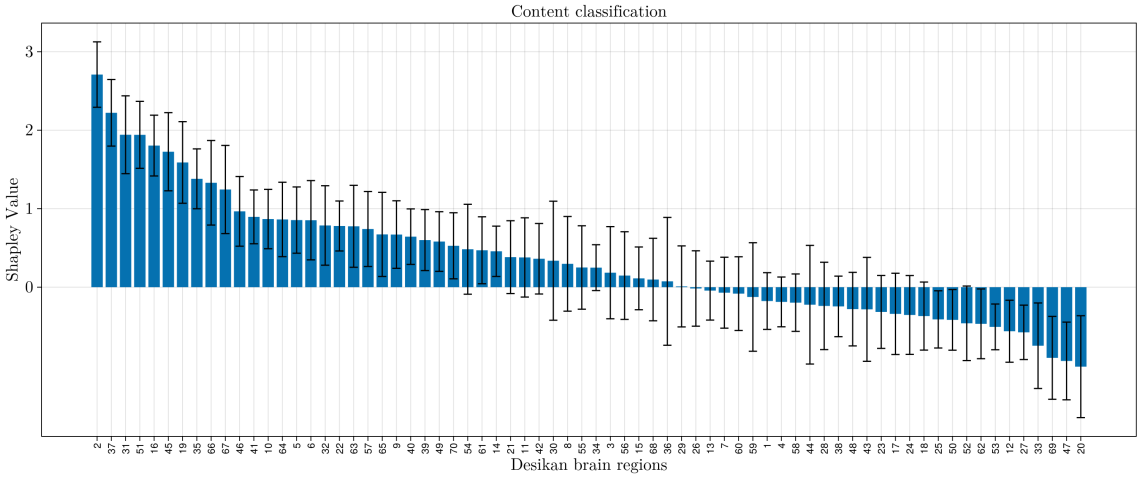

(b)

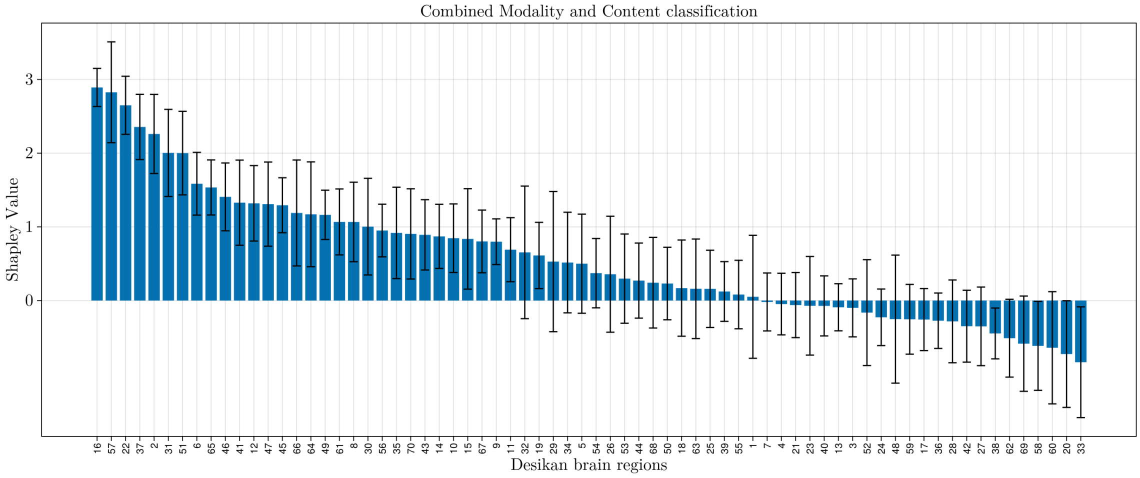

(c)

Figure 2: This figure shows the contribution of 70 Desikan regions, computed using Shapley values, for classifying narratives with our machine learning model. The bars represent the average contribution of each region to the model's predictions, with higher values indicating greater influence. The error bars denote the standard deviation of the Shapley values. The correspondence between label and region can be found in the Table 1

- Intra-subject standard deviation: 37.74%
- Inter-subject standard deviation: 9.21%
- Total variability: 38.92%

We observed that the intra-subject variability is notably higher compared to the inter-subject variability. This disparity could be attributed to the larger number of subjects (31) relative to the smaller number of samples per subject (16).

1.5 Yeo parcellations black and white compatible

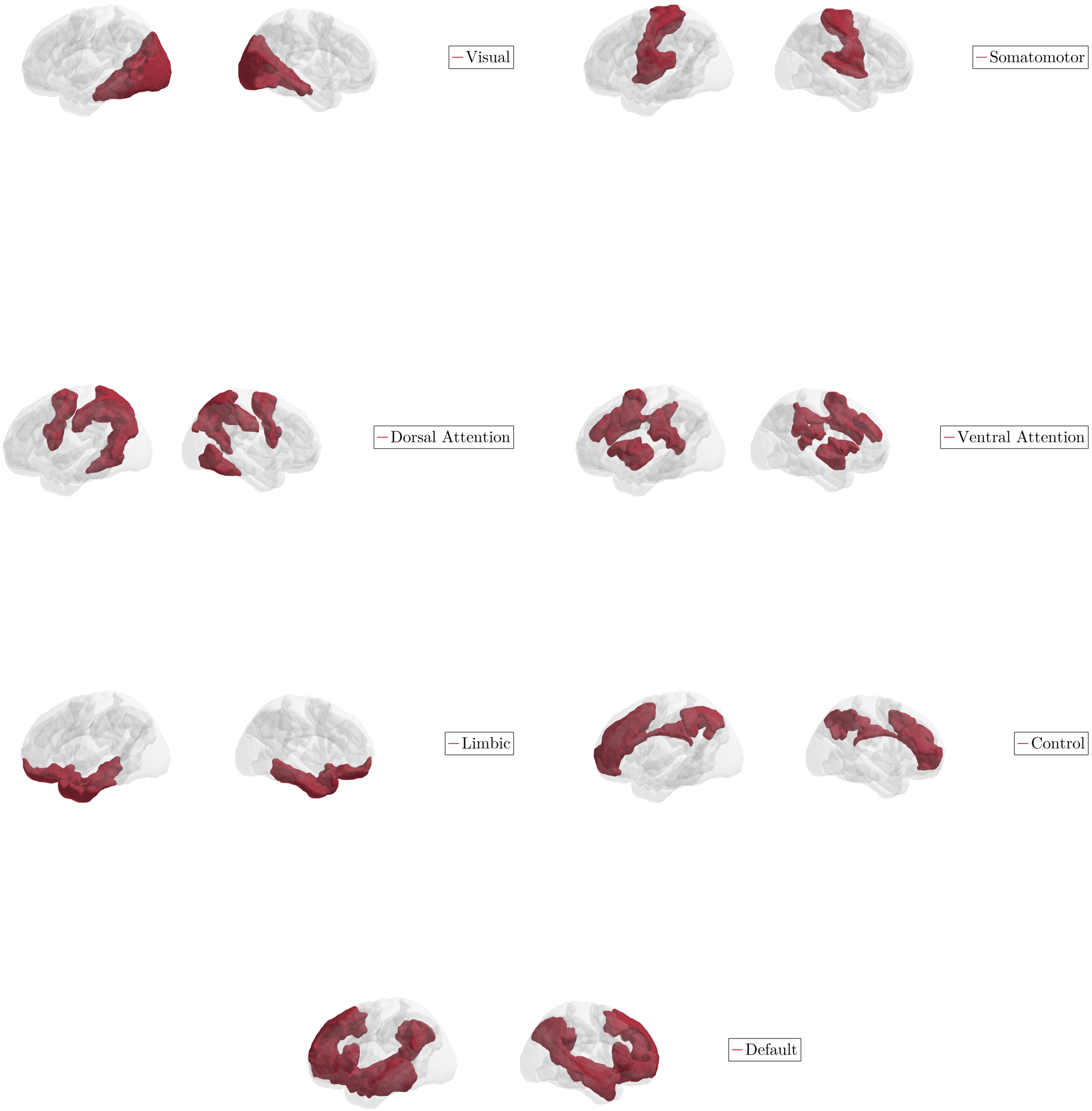

Figure 3: The Yeo 7-subnetwork parcellation illustrates seven distinct functional networks within the brain, each associated with specific cognitive functions. The regions shown in red correspond to areas included in each subnetwork, while the rest of the brain remains in grayscale for contrast. Each pair of images shows the subnetwork from different angles to highlight the distribution of each functional network across the brain. This figure is optimized for black-and-white printing, with clear contrast to make the subnetworks easily distinguishable.

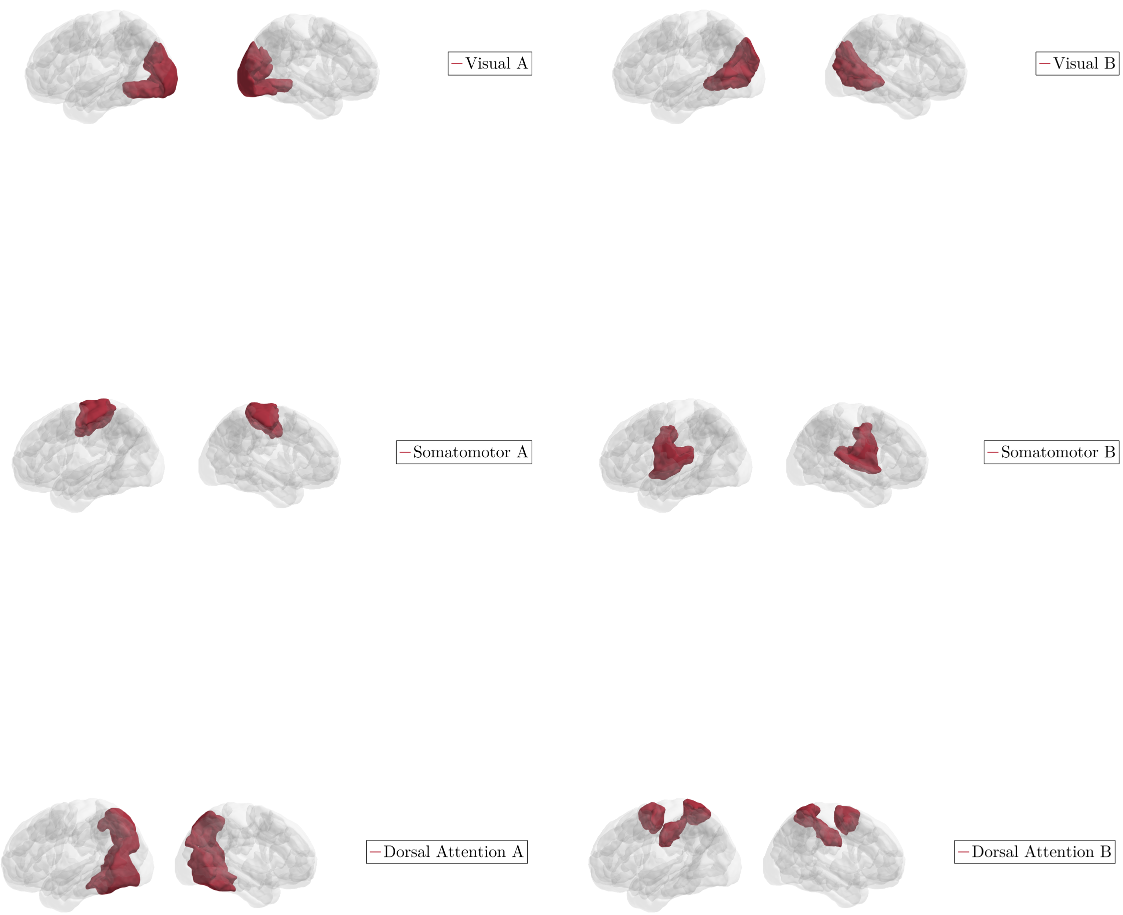
Visual A
Visual B
Somatomotor A
Somatomotor B
Dorsal Attention A
Dorsal Attention B

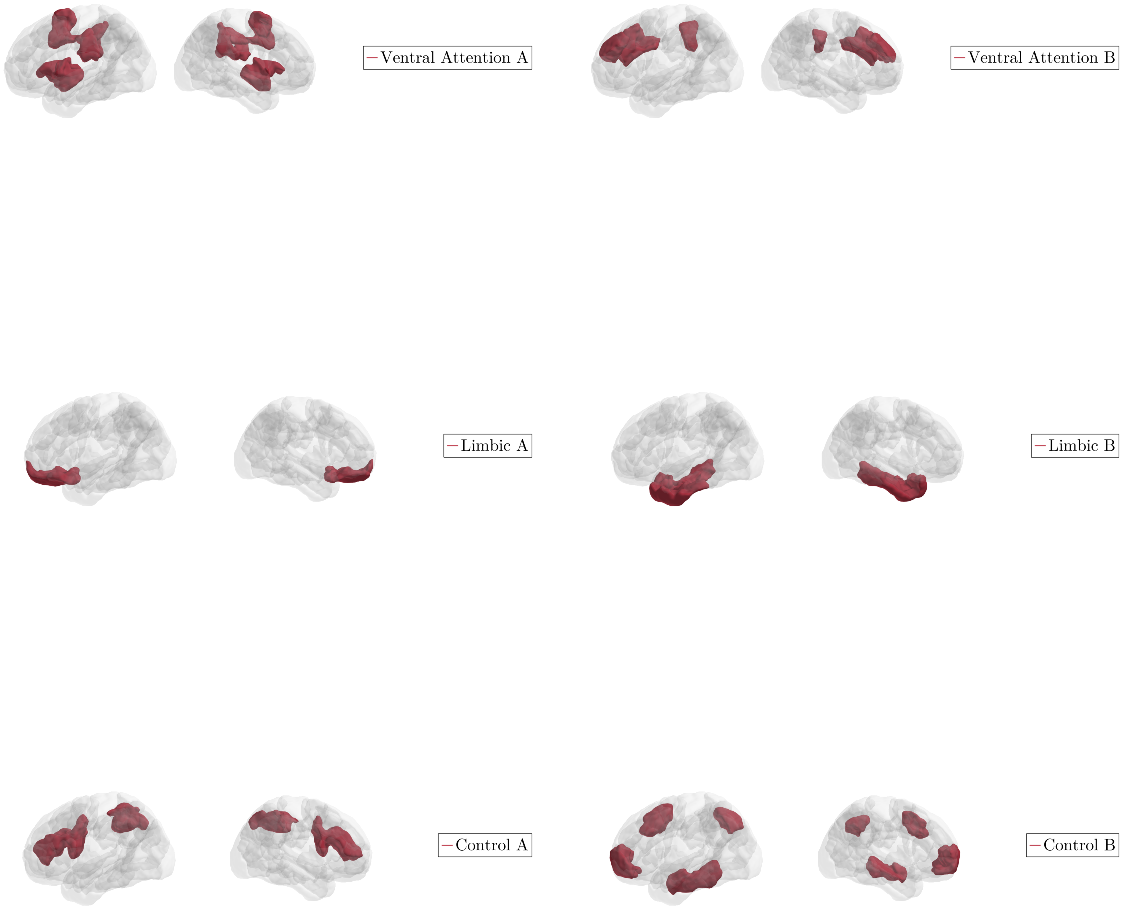
Ventral Attention A
Ventral Attention B
Limbic A
Limbic B
Control A
Control B

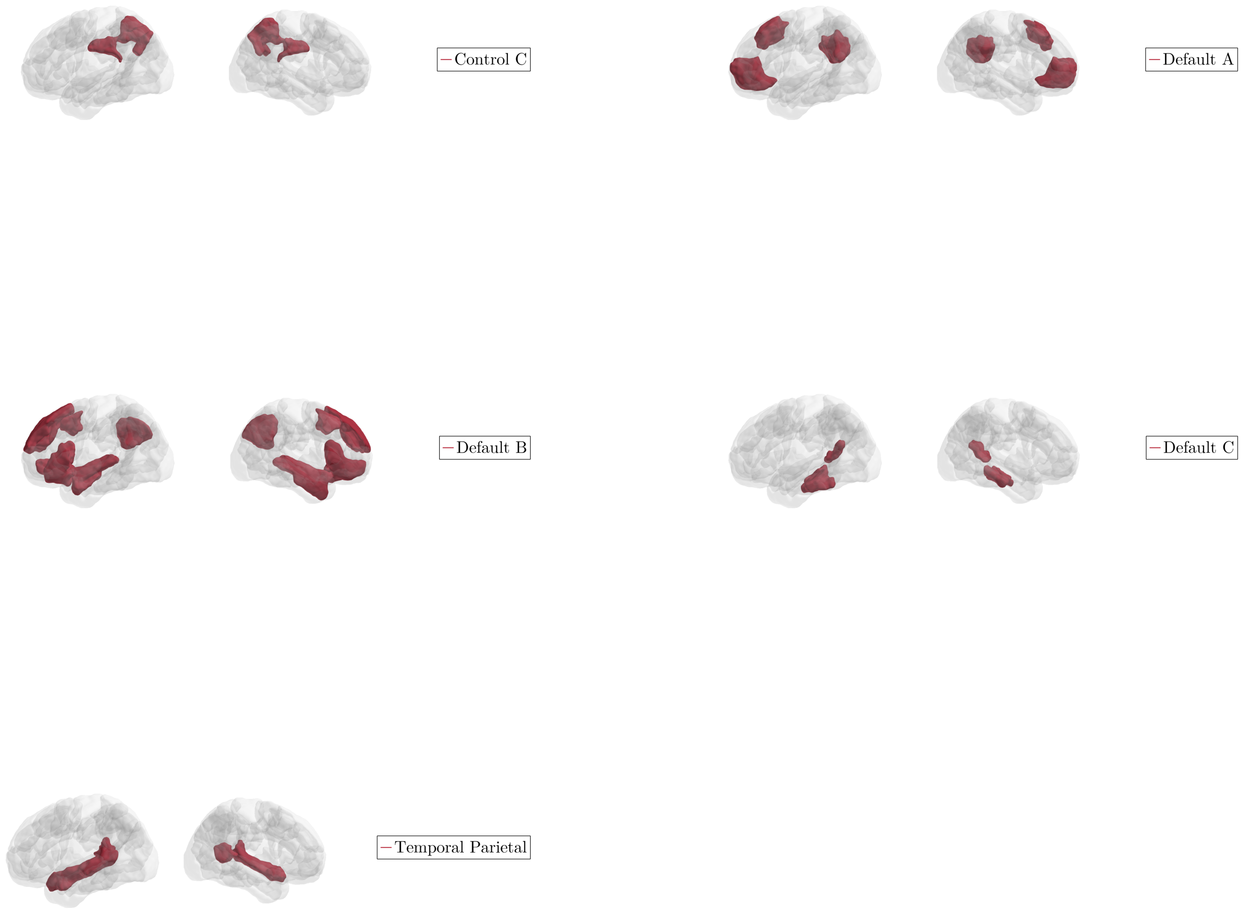

Figure 6: The Yeo 17-subnetwork parcellation illustrates seventeen distinct functional networks within the brain, each associated with specific cognitive functions. The regions shown in red correspond to areas included in each subnetwork, while the rest of the brain remains in grayscale for contrast. Each pair of images shows the subnetwork from different angles, highlighting the distribution of each functional network across the brain. This figure is optimized for black-and-white printing, with clear contrast to make the subnetworks easily distinguishable.
